# Radiation-Grafting on Polypropylene Copolymer Membranes for Using in Cadmium Adsorption

**DOI:** 10.3390/polym15030686

**Published:** 2023-01-29

**Authors:** Rania F. Khedr

**Affiliations:** Chemistry Department Al Leith, University College, Umm Al-Qura University, Mecca 24382, Saudi Arabia; rfkhedre@uqu.edu.sa

**Keywords:** PP films, graft copolymerization, removal of Cd(II)

## Abstract

Graft copolymerization has been a popular technique in recent years for adding different functional groups to polymers. In our research, polypropylene (PP) films are grafted with acrylonitrile (An) and acrylic acid (AAc) monomers to make them hydrophilic while retaining their mechanical qualities. Gamma radiation is used in this approach to establish active spots on an inert polymer that are appropriate for adding monomers radicals to form grafts, a procedure that is extremely difficult to perform using normal chemical processes. The graft parameters are investigated in order to acquire the highest percentage of graft. FTIR (Fourier transform infrared spectroscopy) spectra are used to analyze the grafting of AAc and An. SEM (scanning electron microscopy) and XRD (X-ray diffraction) micrographs are used to validate them. The specimens’ tensile strength and hardness are measured and contrasted with blank PP films. Measurements are made of the effects of grafting on the tensile strength and elongation of the films, and a crucial grafting degree is established in order to preserve these properties. Water uptake is measured to adapt the copolymer to water treatment, and thermal behavior TGA (thermal gravimetric analysis) and DSC (diffraction scanning calorimeter) of the produced copolymer were performed. The elimination of cadmium was verified by an atomic absorption spectrophotometer (AAS) under different conditions of pH, time, and degree of grafting.

## 1. Introduction

Cd(II) could enter aquatic environments through the discharge of untreated wastewater from a variety of industrial operations, including mining, phosphate fertilizers, pigments, alloy industries, electroplating, cadmium and nickel batteries, and sewage sludge [1,2,3,4,5]. The International Agency for Research on Cancer has classified cadmium as being very hazardous and a human carcinogenic [6]. The liver and kidney are the main sites where cadmium builds up [7]. The bones, heart, pancreas, testicles, and hematopoietic system are additional body organs that are exposed to cadmium. Chronic exposure to cadmium has an impact on these organs, impairing their activities and resulting in anemia. Additionally, it can result in bone deformities, lung issues, kidney stone formation, behavioral problems, and cognitive impairment [8,9]. Cadmium toxicity is well known to be the cause of Itai-Itai illness. Therefore, 0.003 mg/L is the maximum allowed level of cadmium in drinking water. The removal of heavy metal ions from wastewater is important for water safety. A lot of absorbents have been developed for this goal. Some of the typical references that focus on different absorbents are suggested to be cited for general readers.

Depending on the sorts of pollutants present in wastewater, different adsorbents have been employed to treat a variety of wastewater types [10,11].

In the past several decades, there has been a remarkable increase in the creation of polymeric selective materials, including membranes with unique functionalities and modification techniques to produce structures suited for use in various separation processes.

These recently developed functional materials come in a number of different physical forms, such as membranes, resins, hydrogels, and adsorbents, which span a range of separation disciplines. The authors of [12] conducted a thorough analysis of the numerous techniques for creating functional membranes and their uses.

Functional copolymers made by radiation-induced graft copolymerization (RIGC) of polar or nonpolar monomers onto existing polymers are a desirable class of separation materials because they have improved properties from the addition of hydrophilic ionic groups that significantly alter the backbone polymers. Additionally, the advantages of radiation-grafted copolymers include (i) easy fabrication; (ii) access to a variety of radiation processing sources; and (iii) the capacity to combine two incompatible characteristics, namely hydrophilicity and hydrophobicity [13,14,15].

Grafted polymers have received a lot of interest recently from researchers looking for an alternative heavy metal adsorbent. Functional monomers are “grafted” onto the polymer chain’s backbone via covalent bonds. Graft polymerization enables the parent polymer to retain its mechanical characteristics while incorporating a variety of functionalities from the grafted monomer [16,17,18].

The radiation-induced grafting technique is superior to other methods for initiating graft copolymerization, such as ionizing radiation, ultraviolet light, plasma treatment, the breakdown of chemical initiators, the oxidation of polymers, etc. because it penetrates deeply into the polymer matrix and produces radicals quickly and uniformly [19,20,21,22,23,24,25].

The aim of this work is to synthesize functionalized membranes that exhibit good properties of swelling and thermal stability and mechanical properties for possible practical applications in the recovery of Cd(II) by complexation from aqueous systems under different conditions of pH, time, and degree of grafting. The use of grafted polymeric materials for the removal of Cd(II) from wastewater has continued to attract considerable attention in recent years because they are capable of binding heavy metals by absorption and ion exchanges even at higher temperatures.

In this research, radiation-induced graft copolymerization of acrylonitrile (AN) and acrylic acid (AAc), both individually and as comonomers, onto polymeric films of polypropylene (PP) was conducted. Varieties of functional groups, such as hydroxylamine, will be introduced into the graft copolymer (cation exchange membrane). So, the possibilities of their practical uses in wastewater treatment by the selectivity of some toxic metal ions i.e., Cd(II) are investigated.

Additionally, some investigations were conducted on the characteristics of the polymeric synthesized membranes and their applications, such as mechanical analyses, thermal analyses, XRD, and SEM, which showed good results [26,27,28,29,30,31,32].

## 2. Experimental Procedure

### 2.1. Materials

PEMEX polypropylene sheets (PP, Mexico City, Mexico) with a thickness of 60 μm and a width of 195 cm in size were rinsed in methanol for 5 h before being dried in a vacuum oven overnight to reach a constant weight. Acrylic acid (AAc) and acrylonitrile (An) were provided by Aldrich(St. Louis, MO, USA). Baker(Radnor Township, PA, USA) provided toluene and methanol, which were purchased and utilized as received.

### 2.2. Procedure of Graft Copolymerization

The grafting of the AAc/An binary system onto the PP by a direct method was achieved first to remove any chemicals adsorbed on the PP surface. The membranes (PP) were washed with acetone two times for 3 min and dried in an oven at 30 °C for 5 h before the initial mass was determined. The initiator FeCl_3_ was dissolved (2.5% by weight) in a combination mixture of distilled water and methanol (with concentrations of 70% and 60% by weight, respectively). This solution was mixed with a solution of monomer mixture AAc/An, and a polymeric strip with this combination was placed in test tubes. Then, degassing with pure nitrogen gas was performed for 5 min, followed by irradiation with Co^60^ Gamma.

The grafted films were removed and washed in acetone and distilled water to eliminate any remaining monomer or homopolymer that had collected in the film, before being dried in an oven for 24 h at 50–70 degrees Celsius. The percentage increase in weight as a result of grafting was obtained by determining the degree of grafting, as follows. The degree of grafting (Dg) was determined using the equation Dg (%) = [(Wg − W0)/W0] × 100, in which Wg is the weight of the grafted fibers and W0 is the initial weight, where Wo and Wg represent the weight of the initial and grafted films, respectively [33].

### 2.3. Preparation of the Cation Exchange Membrane

The grafting monomer used for heavy metal absorption was acrylonitrile, and the graft polymerization yield was kept at roughly 100%. The cyano group of the produced membranes was treated with 3% hydroxyl amine (HO-NH_2_) in a methanol—water combination (1:1) and KOH until pH 7 at 80 °C for 2 h to convert the cyano group of the grafted chains to the amidoxime group (-C(NH_2_)=NOH). As a result, it might be employed as a cation exchange membrane to remove heavy metals. To generate a chelate molecule, the amidoxime group captures metal ions. One heavy metal ion is trapped by two amidoxime groups in the chelate model. As a result, the amidoxime preferentially absorbs heavy metal ions. Because the chelate component releases the metal ion as the pH of the solution changes, the absorbent can be reused [33].

### 2.4. Determination of the Membrane Swelling

The grafted and treated membranes were submerged in distilled water to determine their weight for various degrees of grafting and after being taken out of the water at various intervals until 24 h. They were then weighed and bottled using absorbent paper to remove the droplets on the surface. The following equation was used to compute the value of the water uptake for each degree of grafting [31]:

(1) $$\mathrm{Water}\;\mathrm{uptake}\;\%=\frac{\mathrm{Ws}-\mathrm{Wd}\;\times 100}{\mathrm{Wd}}$$

where Wd and Ws represent the weights of the dry and wet membranes, respectively.

### 2.5. Infrared Spectroscopy

FTIR spectra were collected for both the original and grafted films between 400 and 4000 cm^−1^ with an FT-IR 6300 (Jasco, Tokyo, Japan).

### 2.6. Diffraction of X-rays (XRD)

At room temperature, measurements of X-ray diffraction (XRD, Munich, Germany) were made on both the generated polymers and the blanks. The XRD pattern was captured on the Philips Pw 1730 in the diffraction angle 2 range. The X-ray generator has a built-in scintillation counter. The patterns were produced by diffraction via a nickel filter. (CuKα) λ = 1.45 Å. The X-ray diffractogram was produced under the following experimental conditions: filament current = 28 mA, voltage = 40 kV, and scanning speed = 20 mm/min.

D = Kλ cos θ/B_1/2_

where D denotes the particle size, K 0.89 is the Scherer constant related to the crystal shape and index, CuK, Kλ 1.54056 denotes the X-ray wavelength, cos θ is the diffraction angle, and B_1/2_ is the full width at half-maximum (FWHM in radian) [34].

### 2.7. Scanning Electron Microscopy

Using an energetic electron beam, a JEOL-JSM-5400 scanning electron microscope (Tokyo, Japan) was used to investigate the morphology of the original polymers at high magnification and resolution.

### 2.8. Thermal Gravimetric Analysis (TGA)

A model TGA-50 Shimadzu thermogravimetric analyzer (produced in Kyoto, Japan) was used for the thermal gravimetric analysis measurements (TGA). The nitrogen flow in this investigation was kept constant at around 20 mL/min, and the heating rate was 10 °C/min up to 600 °C, in order to prevent the oxidation of the polymer sample.

### 2.9. Applications (Wastewater Treatment)

Through the absorption of atoms, it is possible to detect trace metals in liquids using a spectrophotometer (AAS), an elemental analysis technique. It is carried out in the presence of other elements by the absorption of radiant energy by atoms at specific wavelength characteristics, and is essentially unaffected by the molecular structure of the metal in the sample, resulting in transitions from a lower to a higher energy state, and this depends on the concentration of the metal. An A-A scan-4 was used for the analysis (AAS), and the produced membranes were tested for the sorption of Cd(II) ions from the water. A total of 20 mL (referred to by V) of the solution (1:5 ppm) of the ions was used and added to 0.1 gm (referred to by m) of the grafted membrane and the % sorption was estimated at different time intervals using the following equation:

(2) $$\%\mathrm{Sorption}=\frac{\mathrm{Ai}-A}{\mathrm{Ai}}\times 100$$

where Ai and A are the initial and final concentrations in mg/L in the solution, respectively.

## 3. Results and Discussion

### 3.1. Grafting Results

#### 3.1.1. Effect of the Solvents and Air Atmosphere

Table 1 illustrates the influence of several solvents on the graft polymerization of An/AAc comonomers onto PP, including H_2_O, methanol, benzene, and a combination of methanol/H_2_O in a nitrogen atmosphere. It was discovered that diluting the monomer mixes with methanol/H_2_O (30:70 wt%) results in a significant improvement in the grafting yield and decreases the homopolymerization process. Because methanol/water is a polar combination, it exhibits a surprising total dissolving of the inhibitor, resulting in minimal homopolymerization. The G-Value (radiation chemical output of free radicals per 100 electron volts absorbed in the system) of the monomer utilized will also be reduced by the MeOH/H_2_0 solvent by a chain transfer reaction by the hydrogen radicals generated. Excess radicals were captured (low G-Value inhibits homopolymer formation during graft polymerization), resulting in a high grafting yield.

#### 3.1.2. Impact of the Inhibitor Concentration

An inhibitor is classified as a radical scavenger because it produces radicals during the initiation phase. These radicals are responsible for controlling the excess of free radicals produced by each monomer, preventing homopolymerization at the optimal concentration of the inhibitor.

**Figure 1:**
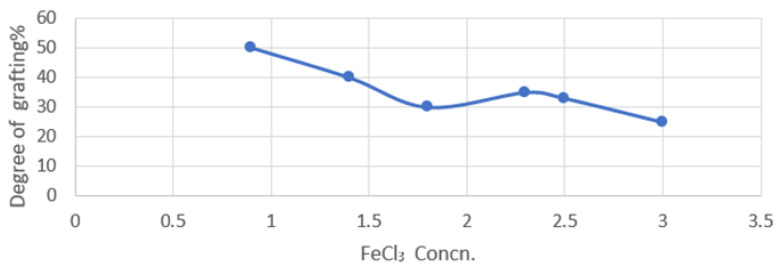
Effect of the concentration of the FeCl_3_ inhibitor (g/L) on the An/AAc grafting degree on the PP films. 30% methanol and 70% H_2_O were used as the solvent in a nitrogen system. The comonomer concentration was 20% by wt% and the comonomer composition was 50:50 by wt% at an irradiation dose of 20 KGY.

The results presented in Figure 1 indicate that the presence of FeCl_3_ improved the grafting process at 2.5% by wt% ferric chloride salt, followed by an effective decrease at a higher concentration. This may be due to the comonomer solution’s higher diffusivity into the bulk polymer.

#### 3.1.3. Effect of the Irradiation Dose

**Figure 2:**
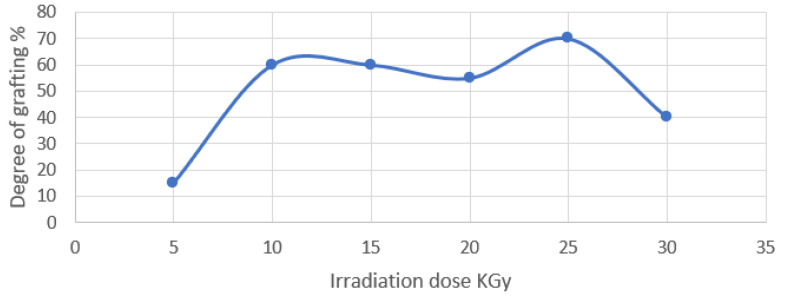
Irradiation dose impact on the polypropylene at an inhibitor concentration of 2.5 gm FeCl_3_ by wt%, at a comonomer composition of 80/20 wt%, at a comonomer concentration of 60%, and a solvent concentration of 70% methanol and 30% H_2_O.

It is well known that raising the irradiation dosage increases the grafting percent by increasing the concentration of free radicals generated either in the polymer substrate or in the monomer solution, resulting in the grafting reaction or cross-linking formation. Figure 2 demonstrates that the grafting yield of the An/AAc monomer combination onto the PP films rises linearly with the irradiation dosage, with a greater grafting yield for the films, perhaps due to differences in morphology. However, the highest grafting yield is reached at 25 KGy, after which the yield steadily declines with the dosage, eventually leveling off at doses greater than 25 KGy. This is due to an increase in the formed radicals (active sites) with the dose (which levels off at higher doses due to recombination) and a restriction in the diffusivity of the An/AAc monomers through the PP films due to the formation of homopolymers at much higher doses [35,36].

#### 3.1.4. Effect of the Comonomer Composition

**Figure 3:**
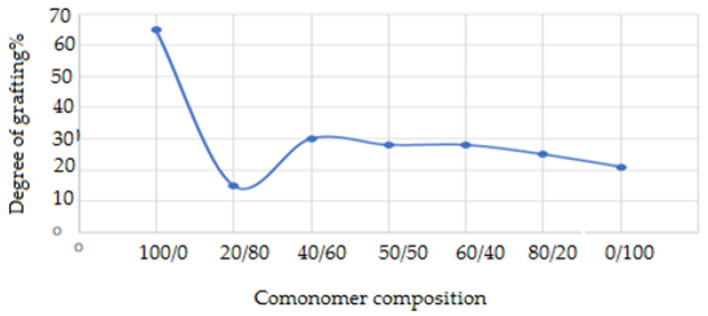
Effect of the comonomer composition on the degree of grafting on the polypropylene at an inhibitor concentration of 2.5 gm FeCl_3_ by wt%, at a comonomer concentration of 60 wt%, and a solvent concentration of 70% methanol and 30% H_2_O, irradiated at 20 KGy in a nitrogen system.

Figure 3 shows the effect of the comonomer composition on the degree of grafting on polypropylene. The grafting yield with AAc in the commoner feed solution increased initially, reaching a maximum degree of grafting at 40/60 An/AAc. The grafting of an An and AAc binary combination onto the PP films has a synergistic impact, according to the findings. The An/AAc ratio in the comonomer mixture determines the amount of the synergistic action. When the An/AAc composition ratio (40/60) wt% is employed for grafting onto PP films, the impact is more prominent. This is owing to the morphological differences between the two films. As a result, it is worth noting that the degree of synergy is determined by the relative proportions of An and AAc in the grafting system [37].

#### 3.1.5. Monomer Concentration

**Figure 4:**
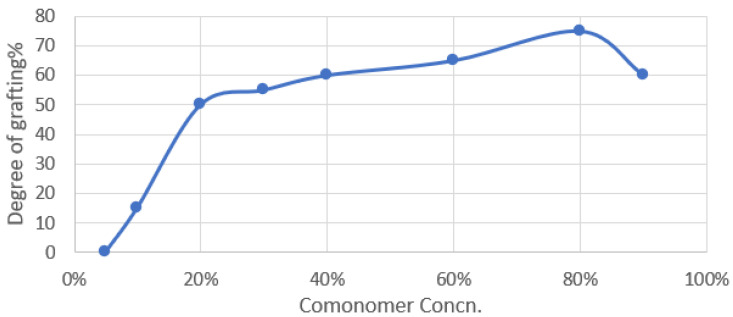
Effect of the comonomer concentration on the degree of grafting on polypropylene at an inhibitor concentration of 2.5 gm FeCl_3_ by wt%, at a comonomer composition of 80/20 wt%, and at a solvent concentration of 70% methanol and 30% H_2_O, irradiated at 20 KGy in a nitrogen system.

Figure 4 shows the effect of a binary monomer combination (An/AAc) with a composition of 40/60 wt% for PP on the grafting copolymerization process. A maximum grafting yield at a certain commoner concentration has been observed in numerous grafting methods. This has been explained by the fact that when the monomer transforms into a polymer, the viscosity of the grafting medium rises. More than mutual termination, the propagation of two growing macro radical chains is hampered. Two lengthy, slow-moving chains are involved in the termination reaction, which might expand. One such long chain and mobile monomer molecule are involved in the propagation process. Due to the development of solid homopolymers at high concentrations of the commoner, propagation tends to decrease while termination rises, lowering the grafting yield. This explains why the grafting yield is highest compared to the commoner concentration.

### 3.2. Grafted Membrane Characterizations

#### 3.2.1. Swelling Patterns

**Figure 5:**
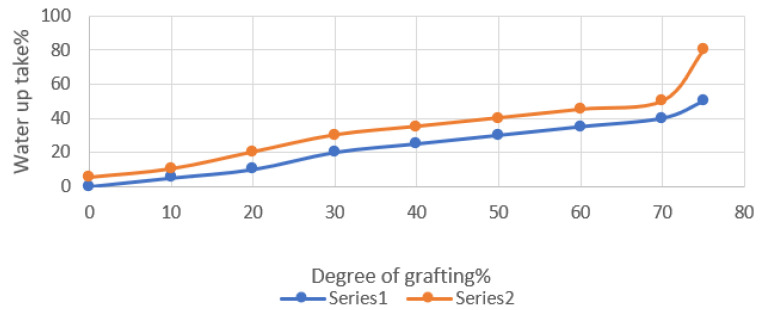
Effect of the degree of grafting on the swelling behavior of PP-g-An-AAc before and after NH_2_OH treatment. (Series 1) grafted, (Series 2) treated.

Figure 5 shows the fluctuation in the membrane water absorption with the degree of grafting of PP-g-p-(An/AAc) for the PP films. According to the findings, the water intake in terms of the weight increases as the degree of grafting increases, reaching a maximum water content of 35 percent for the PP-grafted films. Polyacrylonitrile’s swelling behavior is known to be bad due to its hydrophobic nitrile group (-CN), which was replaced with an amidoxime group. As a result of this chemical reaction, a portion of the grafted copolymer membrane is changed to an absorbent agent. The treated membranes’ water absorption has increased significantly. This may be due to the nitrile groups being converted to amine groups, which improves the swelling behavior of the grafted films, which is dependent on the degree of grafting and the functional groups.

#### 3.2.2. Fourier Transform Infrared Spectroscopy (FTIR)

**Figure 6:**
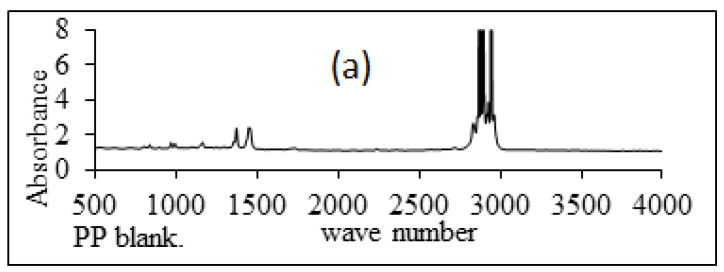

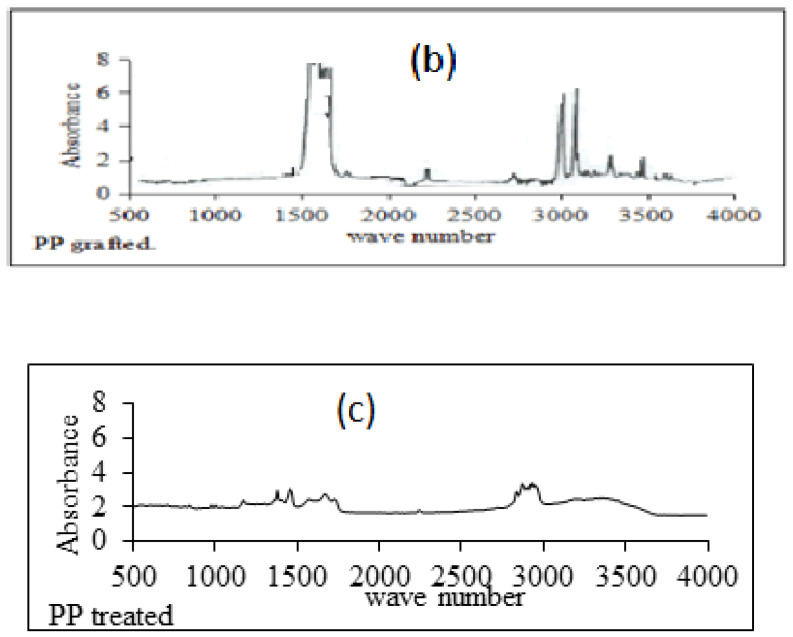
FTIR spectra for polypropylene. (**a**) pure, (**b**) grafted, (**c**) treated.

An infrared spectroscopic examination of PP films grafted with a comonomer combination of An/AAc and treated with hydroxylamine in an aqueous NaOH solution to convert the hydrophobic (C=N) group in P(AN) to a hydrophilic amidoxime group was conducted. The use of IR analysis was utilized to track this changeover. Figure 6a–c shows the IR spectra of the PP blank, grafted, and amidoxaminated membranes, respectively. As a consequence of amidoxamination, it is obvious that the absorption band for C=N is about 2200 cm^−1^. As a result of amidoxamination, it is obvious that the absorption band for the C=N group, which reduced significantly but did not disappear completely, disappeared totally at roughly 2200 cm^−1^. This is because the membrane did not swell sufficiently in the aqueous media before the amidoxamination reaction began to improve and swell effectively with conversion as a result of the hydrophobic nitrile group being replaced (C=N) with the hydrophilic amidoxime group (N=OH), which appears on the spectra at a broad band at 3200–3500 for the PP membranes.

#### 3.2.3. Mechanical Measurements

**Figure 7:**
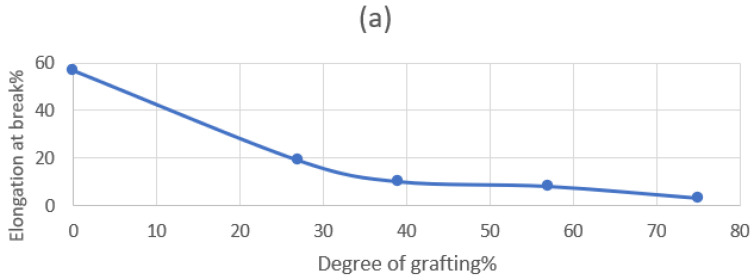

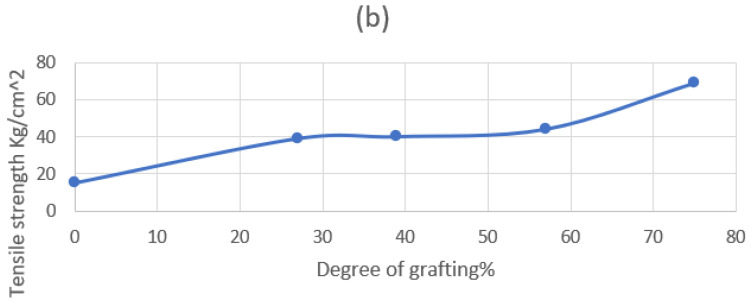
Effect of the degree of grafting on (**a**) the tensile strength and (**b**) the % elongation at the break on PP.

As shown in Figure 7a,b, it is found the relation is inverse between the tensile strength and the degree of grafting; however, the elongation has a proportional relationship with the degree of grafting. This can be explained according to the rigidity of the surface increase as the degree of grafting increases, so there is an increase in the tensile strength and a reduction in elongation. Thus, for different applications in wastewater treatment, it is important to obtain a high tensile strength for the treated grafted membranes.

#### 3.2.4. Thermal Gravimetric Analysis (TGA)

**Figure 8:**
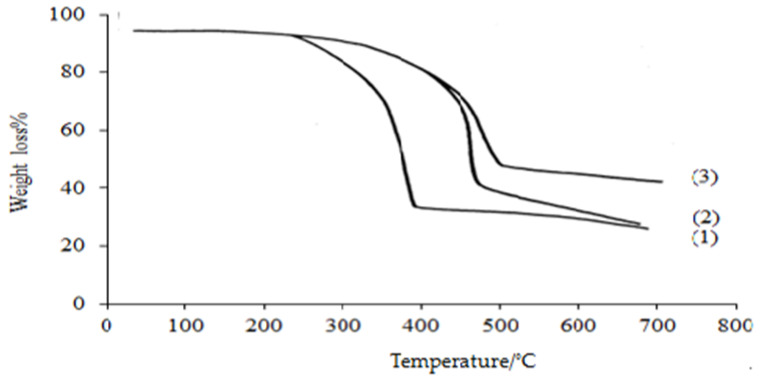
Thermal gravimetric analysis for the polypropylene samples. 1—PP blank, 2—PP-grafted, 3—PP amidoximated.

Figure 8 (1) shows the PP blank membranes are stable up to 400 °C and start to decompose around this temperature with a weight loss of about 80 to 90%. However, in Figure 8 (2,3), the pp-g-p(An/AAc) and amidoxaminated membranes start to decompose at about 460 °C and 500 °C, respectively, above this temperature with an observed rapid weight loss of about 30% and 32%, respectively. This is due to the introduction of the graft chains of An/AAc seen to be cross-linked and the presence of a thermally stable amidoxime group [38].

#### 3.2.5. Differential Scanning Calorimeter (DSC)

**Figure 9:**
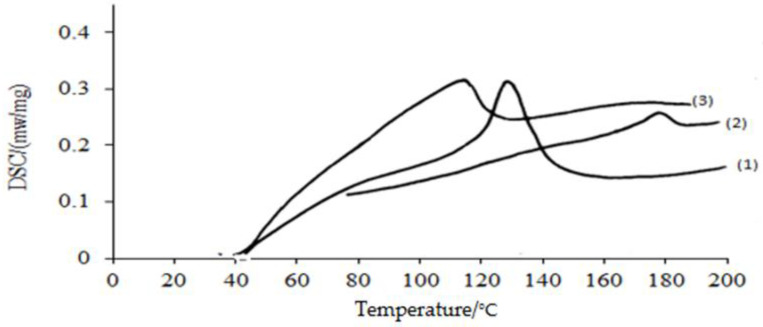
Differential scanning calorimeter diagram for the polypropylene samples. 1—PP amidoximated, 2—PP-grafted, 3—PP blank.

It can be seen in Figure 9 (1–3) that the crystalline melting peak temperature for the PP blank was 110 °C, for the grafted PP was 130 °C, and for the treated PP was 180 °C. It can also be seen that the crystalline melting peak temperature slightly shifted to low temperatures for the grafted and treated films. As a result of the disordering and cross-linking caused by the grafting and amidoxamination onto the PP membranes, which is the cause of the chain mobility limitation, this modification indicates that the grafting causes changes in the crystalline areas.

#### 3.2.6. Scanning Electron Microscopy (Morphology)

**Figure 10:**
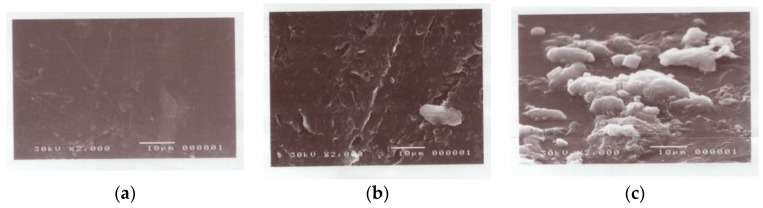
Scanning electron micrographs of the PP membranes before and after grafting. (**a**) PP blank, (**b**) the PP-grafted membrane, and (**c**) the PP amidoximated membrane.

Figure 10a–c shows the morphologies observed by SEM of the PP blank and the grafted and amidoximated (treated with hydroxyl amine) films. The surface texture of the PP blank appears with no spots as there is not any reaction on the surface; however, in the grafted PP, there are spots in the surface texture, and the amidoximated PP shows a lot of spots, grooves and no homogeneity in the surface texture due to the reaction of the functional groups of An/AAc with NH_2_OH.

#### 3.2.7. X-ray Diffraction (XRD)

**Figure 11:**
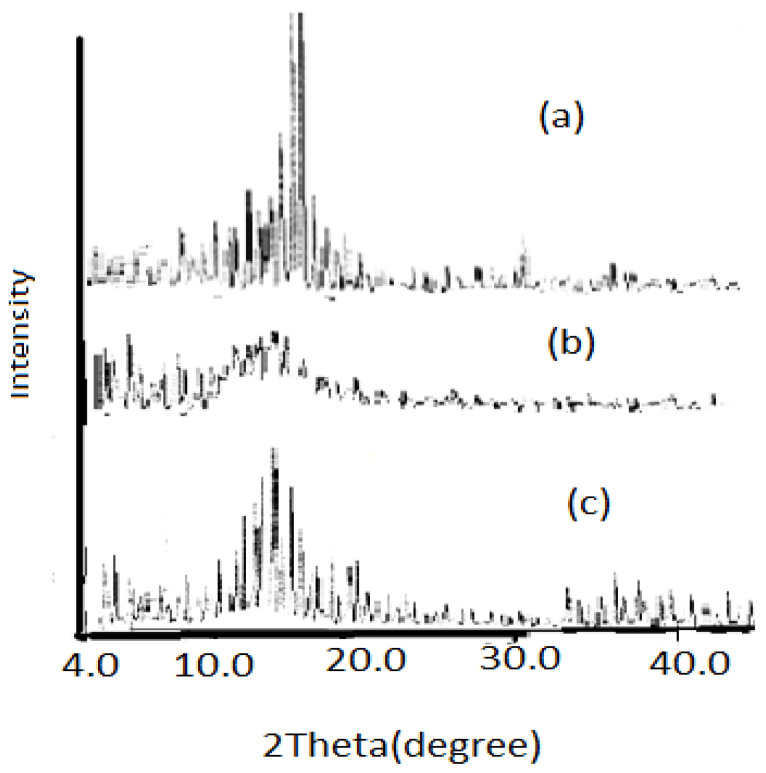
XRD pattern for PP. (**a**) blank, (**b**) grafted, and (**c**) amidoxaminated.

Figure 11a–c demonstrates an XRD diffractogram pattern for the PP blank, PP-g-p(An/AAc), and amidoxaminated membranes, respectively. The figures indicate that the crystalline structure is different in each case according to the intensity of the main diffraction line, which shows the decrease in the crystallinity of the grafted PP and treated PP membranes, and that is due to a change in the amorphous region of the grafted and amidoximated membranes, which results in disorder in the structure [39].

### 3.3. Application in Wastewater Treatment for the Removal of Cd(II)

#### 3.3.1. Effect of the Grafting Degree

**Figure 12:**
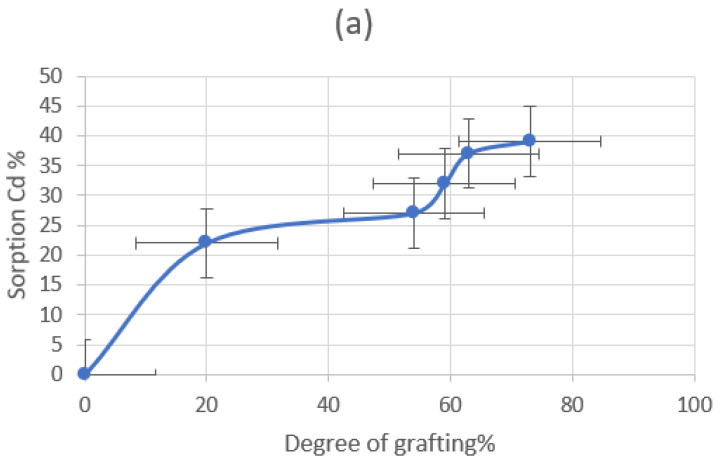

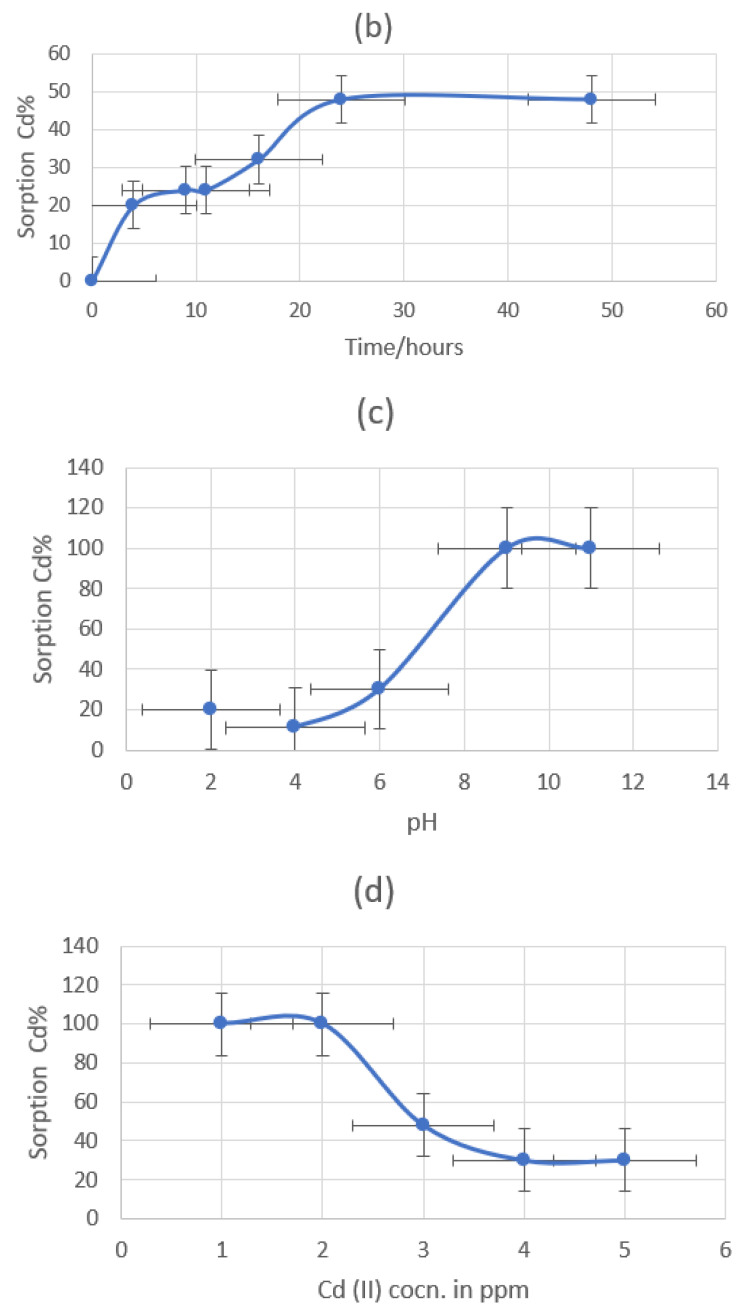
(**a**) Effect of the degree of grafting on the sorption of Cd(II) on polypropylene grafted membranes, (**b**) Effect of the contact time on the sorption of Cd(II) on polypropylene grafted membranes, (**c**) Effect of the hydrogen concentration on the sorption Cd(II) on polypropylene grafted membranes, and (**d**) Effect of the metal ion concentration on the sorption of Cd(II) on polypropylene grafted membranes.

Figure 12a demonstrates how the grafting degree yield affects the elimination of Cd(II) from the PP-grafted and amidoxaminated membranes (PP-gp-An-AAc). It is clear that as the grafting intensity increases, so does the percentage of metal ion absorption. This shows that the elimination or complexation of metals depends on the quantity of the functional reactive groups in the membranes [40].

#### 3.3.2. Kinetics of Sorption

Using Figure 12b as an example, the metal sorption is shown as a function of the contact or treatment time. (PP-g-An-AAc). For Cd^2+^ removal, it is shown that the Cd^2+^ uptake rises until it reaches its maximum point after 24 h, and the sorption of Cd^2+^ depends on different factors, such as the polarity of the metal ion, its electronic structure, and the ionic radius, as well as the type of interaction with the membrane’s functional groups [41].

#### 3.3.3. Effect of pH on Sorption

The pH of the solution depends on both the surface charge of the PP-gp-(An-AAc) and the charge of the Cd(II) and the effect on Cd(II) uptake. The results of the pH variation are presented in Figure 12c, and they indicate that at pH 9, with an accumulation of 80%, the most metal was removed. Figure 12c illustrates the Cd(II) adsorption, which peaks at pH 9 and is related to an increase in the OH-(hydroxyl ions); however, it gradually declines until pH 10, as indicated. Then, a major drop is observed at pH > 10. This might be attributed to the precipitation of Cd(II) at pH∼9.4. Hence, at low pH levels, the hydrogen ion competes with accessible exchange sites, reducing the absorption of Cd(II) cations. After that, as the pH increases, the H^+^ decreases and the proton’s competitive impact on the metal cation diminishes, increasing the percentage of Cd(II) ions that sorb. The presence of ions such as OH and NH^2+^ groups, as well as the high negative surface of the PP-g-p-(An-AAc), which might attract positively charged Cd^2+^, are credited with the material’s capacity to adsorb Cd(II) [39]. So, the results in Figure 12c show that the optimum pH to remove Cd(II) from the solution using PP-g-p-(An-AAc) is 8–10 [42].

#### 3.3.4. Effect of the Metal Ion Concentration

Investigations were performed regarding how the metal ion concentration affected the sorption of Cd(II) by PP-g-p-(An-AAc). The data presented in Figure 12d demonstrated that as the concentration of the metal ions rises so does the sorption up to a specific value (2 ppm), beyond which it falls. This may be explained by the fact that at specific concentrations, the Cd(II) ions saturate the active sites (amidoxime and hydroxyl functional groups), which are responsible for chelation, and there are no viable adsorption sites at very high concentrations since the sorption decreases [43].

## 4. Conclusions

Utilizing the irradiation approach, PP-g-p-(An-AAc) films are synthesized. We employed a solvent combination that included 30% methanol and 70% water under a nitrogen environment and a concentration of the FeCl_3_ inhibitor to avoid homopolymerization (2.5% by weight). The maximum graft yield was 115% with a radiation dosage of 20 kGy, a monomer ratio of 40/60, and an 80% comonomer concentration. To create an amidoxime adsorbent, hydroxylamine hydrochloride was used to modify the An/AAc grafted films. The synthesized adsorbent was evaluated for mechanical characteristics, water absorption percent, FTIR, SEM, DSC, and TGA. A high affinity for Cd(II) adsorption was demonstrated by the produced amidoxime adsorbent. After 48 h of contact time at pH 9 and a starting metal ion concentration of 2 ppm, the greatest adsorption capacity was attained: 100 mg/g.

## Figures and Tables

**Table 1:** Effect of inhibitor concentration (g/L) on the degree of graft of AN/AAC on PP films.

Solvent	PP G%	Comment
H_2_O	166	High homopolymerization
Mixture of 30% methanol, 70% H_2_O	115	Optimum condition
Methanol	22	
Benzene	2	
**Effect of Air atmosphere**	**PP G%**	
Air	38	
Nitrogen	65	Optimum condition

## Data Availability

Not applicable.
